# Effect of Water Storage on Hardness and Interfacial Strength of Resin Composite Luting Agents Bonded to Surface-Treated Monolithic Zirconia

**DOI:** 10.3390/dj9070078

**Published:** 2021-07-04

**Authors:** Emmanouil-George Tzanakakis, Maria Dimitriadi, Ioannis Tzoutzas, Petros Koidis, Spiros Zinelis, George Eliades

**Affiliations:** 1 Department of Operative Dentistry, School of Dentistry, National and Kapodistrian University of Athens, 11527 Athens, Greece; tzoudent@dent.uoa.gr; 2 Department of Biomaterials, School of Dentistry, National and Kapodistrian University of Athens, 11527 Athens, Greece; mardimit@dent.uoa.gr (M.D.); szinelis@dent.uoa.gr (S.Z.); geliad@dent.uoa.gr (G.E.); 3 Department of Prosthodontics, School of Dentistry, Aristotle University of Thessaloniki, 54124 Thessaloniki, Greece; pkoidis@dent.auth.gr

**Keywords:** zirconia ceramic, resin composite luting agents, sandblasting, shear bond strength, hardness

## Abstract

Background: Durable bonding between resin composite luting agents (CLA) and zirconia is still a matter of controversy. The purpose of this study was to evaluate the effect of water storage on hardness and interfacial strength of three CLA, a non-adhesive (Multilink Automix/ML), an adhesive (Panavia F 2.0/PF) and a self-adhesive (PermaCem 2.0/PC), bonded to polished (CL) and grit-blasted (AL: 50 μm alumina, SJ: Sil-Jet + Monobond Plus silane) monolithic zirconia surfaces. Methods: CLA specimens (*n* = 5/cement, condition) were prepared, stored under dry conditions or immersed in water, and Vickers hardness (VH) measurements were obtained at 1 h, 24 h, 1 week and 3 weeks intervals. Optical profilometry was used to determine the roughness parameters (Sa, Sz, Sdr, Sci) of zirconia surfaces (*n* = 5/treatment). A shear strength test (SBS, *n* = 10 × 2/cement) was performed to assess the strength and fractography of the cements bonded to zirconia after isothermal water storage and thermal-cycling (TC). Results: PF demonstrated significantly lower VHN after water storage at all time intervals, PC at 1 w, 3 w and ML at 3 w. SJ and AL showed significantly higher values from CL in all roughness parameters. Weibull analysis revealed the following significance in σ_ο_ ranking within the same material: AL, SJ, ALTC > SJTC, CL > CLTC (PF); SJ, SJTC, AL, ALTC > CL, CLTC (PC) and SJ, SJTC > AL > ALTC > CL, CLTC (ML). Within the same surface treatment subgroups, the significance in σ_o_ ranking was PC, ML > PF (before/after TC) for SJ; PC > PF > ML (before TC), PC, PF > ML (after TC) for AL, and PC > PF > ML (before/after TC) for CL. For the m ranking, the only significant difference within each material group was found in PC (AL > ALTC) and for the same surface treatment in AL (PC > ML). Conclusion: There are significant differences in the water plasticization susceptibility of the CLA tested; the materials with adhesive monomers were the most affected. Tribo-chemical silica coating combined with a silane coupling agent was the most efficient bonding treatment for the non-adhesive and the self-adhesive materials. The adhesive CLA performed better on alumina-blasted than on tribo-chemically coated surfaces.

## 1. Introduction

Excellent mechanical properties, acceptable esthetics and biocompatibility have made 3Y-TZP zirconia popular for all-ceramic restorations [1]. Nevertheless, the best method for a durable bonding between zirconia and tooth structure using resin composite luting agents is still a matter of controversy [2]. Μicro-mechanical retention and chemical bonding are the determinant factors for resin bonding to zirconia. Micromechanical retention is dependent on surface topography, since a rough surface provides an extended and complex area for resin infiltration. Unlike glass-ceramic surfaces, zirconia is very difficult to be effectively etched with HF acid [3]. Several alternative surface treatments (grit-blasting, hot/concentrated acids, glass coatings, laser engraving, etc.) have been proposed to establish a highly retentive zirconia surface, but with contradictory results [2,4]. Grit-blasting, with a clinically proven versatility, has been readily introduced for zirconia roughening. The use of 50 μm alumina particles at low pressure has been indicated as an efficient zirconia surface roughening treatment based on laboratory [5] and clinical studies [6]. This treatment has been considered safe regarding destabilization of the tetragonal zirconia phase [7]. For chemical bonding, adhesive monomers, mainly phosphate functionalized methacrylates, have been introduced in the resin composite luting agents and/or in their primers [3,8,9]. These monomers have been shown to react with zirconia [10,11,12,13] via three mechanisms, producing different Zr-P compounds [14]. The monodentate Zr-P salt has been considered as the primary bonding mechanism between a phosphate monomer (10-methacryloyloxydecyl dihydrogen phosphate/10-MDP) and zirconia [15], although the contribution of the bridging bidentate (two Zr bonded to one P) and phosphates bridged to Zr (two P bonded to one Zr) compounds cannot be excluded [10]. In laboratory studies the use of 10-MDP improved the tensile and shear strength of resin composite luting agents bonded to zirconia [16,17]. An alternative procedure was the use of silanes, which have long established applications in dentistry [18]. Although it has been claimed that silanes cannot react directly with zirconia [19,20], there is experimental evidence that they can react with zirconia powder forming Si–O–Zr bonds, while they may also stabilize the tetragonal zirconia phase [21]. Despite this controversy, silane optimization is achieved by tribo-chemical silica coating of zirconia, as silica enrichment of zirconia surface facilitates bonding by formation of Si–O–Si bonds between the silane and the surface implanted silica particles [22,23].

Recently, efforts have been undertaken to simplify the multistep clinical procedures of resin bonding to zirconia and teeth by introducing single-step self-adhesive resin cements, where acidic methacrylate monomers are incorporated into the paste components of auto-mix systems [23,24]. Although concerns have been expressed about the hydrolytic stability of these cements, they became popular due to their simplified bonding procedure. The development of monolithic zirconia restorations has raised questions about the bonding capacity of resin cements. Increased yttria (up to 5.2%) and reduced alumina contents (<0.5%) are the main structural differences from conventional 3Y-TZP materials used for porcelain veneering [25]. Monolithic restorations demonstrate increased translucency [26] and require different sintering and fabrication processes [27], which may influence their mechanical properties and consequently the outcome of grit-blasting treatments relevant to adhesion.

The purpose of this study was to evaluate (a) the stability of three types of commercially available resin composite luting agents for water plasticization and (b) the effect of two grit-blasting methods on the bond strength of the luting agents to monolithic zirconia before and after aging. The null hypotheses were that (a) all the luting agents are not prone to water plasticization and (b) the type of luting agent, zirconia surface treatment and aging do not influence the bond strength with zirconia.

## 2. Materials and Methods

The resin composite luting agents (CLA) used in study are listed in Table 1. Two materials contain adhesive monomers (PF, PC) and one (ML) does not. PC is a self-adhesive material, PF a two-phase adhesive material with self-adhesive primers and ML a material with self-adhesive primers. For ML and PF, the primers are mainly used for bonding to dental hard tissues. The stability of the CLA to water plasticization was evaluated by hardness measurements (dry vs. water-stored specimens), the effect of grit-blasting methods on zirconia topography by roughness measurements and the bond strength to monolithic zirconia by a shear test.

### 2.1. Hardness Measurements

Teflon molds (diameter = 10 mm, height = 3 mm) were used to prepare the CLA specimens. The molds were placed on microscopic transparent glass plates covered with cellulose strips, filled with the CLA, covered with another set of strips/plates, pressed to remove material excess and stored for 10 min in dark and dry conditions at 37 °C. Then the specimens were light-cured from top and bottom surfaces (2 × 20 s) employing a LED light-curing unit (Radii Plus, SDI, Bayswater, Victoria, Australia) emitting 1.5 W/cm^2^ light intensity in the standard irradiation mode. Two specimen series were prepared from each CLA; the first was stored as before, whereas the second was immersed in distilled water (37 °C). Hardness measurements were obtained at 1 h, 24 h, 1 w and 3 w time intervals (*n* = 5/material, immersion mode and storage period) using a hardness tester (Diatronic 2RC, Wolpert, Ludwigshafen, Germany) equipped with a Vickers indenter under a 1 kp load, 70× magnification and 10 s contact period. Three indentations were made on each specimen in an equilateral triangular mode, 2 mm distant to the margins, and the HV_1kp_ values were averaged.

### 2.2. Roughness Measurements

Cylindrical monolithic zirconia blocks (diameter = 10 mm, height = 8 mm, BruxZir Solid Zirconia HT-2.0, Glidewell, Newport Beach, CA, USA) were prepared. All specimens were embedded in epoxy resin (Epofix, Struers, Ballerup, Denmark), ground in a grinding/polishing machine (DAP V, Struers) at a speed of 200 rpm using silicon carbide papers (220, 240, 400 and 600 grit-size) under water-coolant, ultrasonicated in ethanol for 3 min, water-rinsed and air-dried. The specimens were equally divided into three groups (CL, AL, SJ) and treated as follows. In CL the polished specimens received no further treatment and were used as control. The AL specimens were air-abraded with 50 μm alumina particles using an intraoral sandblaster (Microetcher II A, Danville Materials, S. Ramon, CA, USA) operated for 10 s at 0.25 MPa air pressure, from 10 mm distance and 90° incidence angle. The SJ specimens were subjected to tribo-chemical silica coating with Sil-Jet powder (30 μm alumina/silica particles, Danville Materials) under the same conditions. The 3D-surface roughness parameters of the zirconia specimens (*n* = 10/group) were determined employing an optical profilometer (Wyko NT 1100, Veeco, Tuscon, AZ, USA) under the following conditions: Mirau lens, vertical scanning mode (VSI), 303.8 × 231 μm^2^ analysis area (20× magnification, 0.1 nm (*z*-axis) and 0.2 μm (x- and y-axes) resolution. Two amplitude (Sa, Sz), one hybrid (Sdr) and one functional (Sci) parameters were measured. Sa is the arithmetical mean of the absolute values of the surface deviations above and below the mean plane within the sampling area and represents an overall measure of the surface texture. Sz is the average value of the absolute heights of the five highest peaks and the depths of the five deepest pits or valleys within the sampling area. Sdr expresses the ratio of the increment of the interfacial area of a surface over the sampling area. Finally, Sci is the ratio of the void volume of the unit sampling area at the core zone defined as 5–80% of the bearing ratio [28].

### 2.3. Bond Strength Testing

Zirconia specimens prepared as above were randomly classified into three subgroups (CL, AL, SJ, *n* = 20 each). In SJ all specimens were primed with a phosphate and disulfide containing universal silane (Monobond Plus, Ivoclar Vivadent/MB) according to the manufacturer’s instructions and dried with oil-free air. Split solid Teflon molds with a central bore of 3 mm in diameter and 3 mm in depth were secured at the center of the treated specimens surfaces, filled up with the CLA, covered with an air barrier (Oxyguard II, Kuraray Noritake), stored for 10 min at 37 °C (dark/dry) and then light-cured as before. After removal of the molds, all specimens were immersed in deionized water at 37 °C for one week. Then half the number of specimens of each subgroup were aged by thermal-cycling (TC) in water (5/55 °C, 500× cycles, 20 s immersion time, 10 s transfer time), whereas the rest were aged isothermally in water for the same period. All samples were then loaded at the interface until fracture, utilizing a shear device with a notched-end metallic piston in a universal testing machine (Tensometer 10, Monsanto, Swindon, UK) at a 0.5 mm/min crosshead speed. The de-bonded zirconia surfaces were examined under a stereomicroscope (M80, Leica, Weltzar, Germany) at 25× magnification to assess the failure modes. The de-bonded areas were characterized employing a five score index, based on the percentage area covered by resin over a normal pentagon projected on the field of view (scores 1: 0–20%, 2: 21–40%, 3: 41–60%, 4: 61–80% and 5: 81–100%). Score 1 was considered as an adhesive failure, scores 2–4 as mixed with an increasing resin cohesive character and score 5 as a resin cohesive failure.

### 2.4. Statistical Analysis

The results of the hardness measurements were subjected to two-way ANOVA (material, storage condition and storage period as the independent variables). Nevertheless, since the normality test failed (Shapiro-Wilk *p* < 0.05) the analysis was limited to one-way ANOVA (storage period per condition and material) and t-tests (same storage period between the two storage conditions). The roughness parameters were statistically analyzed by one-way ANOVA plus Least Significant Difference method (LSD) for individual comparisons. For shear bond strength data (SBS), Weibull analysis was performed to determine the reliability and strength of each treatment. For each SBS subgroup the Weibull modulus (m) and characteristic strength (σ_ο_) were determined. Failure modes were analyzed using Pearson Chi-square statistics. The statistical analyses for roughness and failure mode were performed by Statistica 10 software (StatSoft Inc., Tulsa, OK, USA) and the Weibull analysis by Excel software (Microsoft Corp, Redmond, WA, USA) at a 95% confidence level (α = 0.05).

## 3. Results

### 3.1. Hardness Measurements

**Figure 1:**
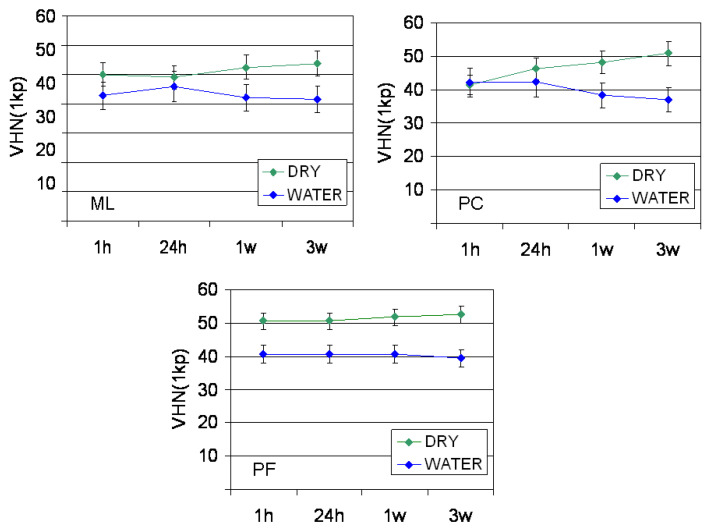
The results of VHN_1kp_ measurements. ML: Multilink Automix, PC: PermaCem 2, PF: Panavia F 2.0.

The results of hardness measurements are presented in Figure 1. For all materials statistically insignificant differences were found in the VHN_1kp_ values between the time intervals tested within the same storage condition (*p* > 0.05). Water storage resulted in a significant hardness reduction in PF (all time intervals, *p* = 0.001–0.005), PC (1 w, 3 w, *p* = 0.008 and 0.016 respectively) and ML (3 w, *p* = 0.026).

### 3.2. Roughness Measurements

**Figure 2:**
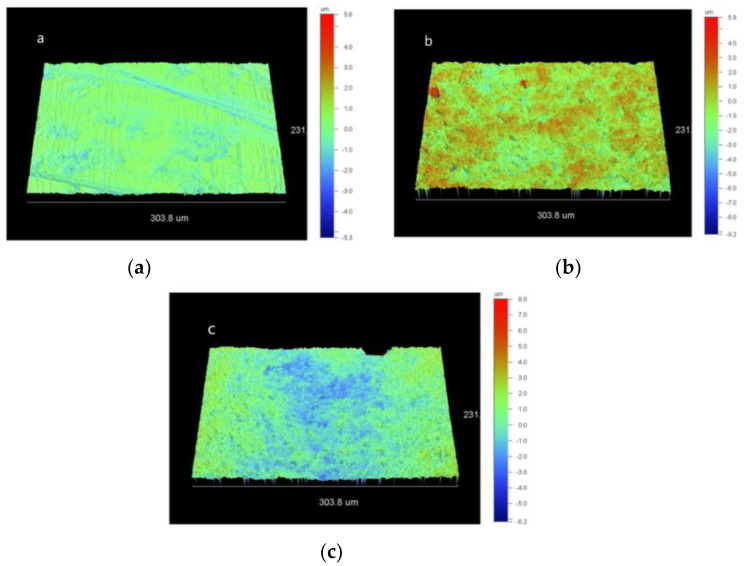
Representative 3D-profilometric images of CL (**a**, scale range: 5.6 to −5.3 μm), AL (**b**, scale range: 5.8 to −9.2 μm) and SJ (**c**, scale range: 8 to −6.3 μm) treated zirconia surfaces (20× magnification).

Representative 3D-profilometric images of the zirconia surfaces per treatment group are illustrated in Figure 2a–c. Polished specimens (CL) exhibited a homogeneous texture with small peaks and shallow valleys, whereas grit-blasted groups (AL and SJ) demonstrated randomly distributed deep valleys and high peaks. The results of the 3D-roughness parameters are summarized in Table 2. For Sa, statistically significant higher values were documented in AL and SJ groups in comparison with CL (*p* < 0.01), but not between AL and SJ (*p* = 0.562). A similar statistical ranking was found for Sz; there were statistically significant differences between SJ–CL and AL–CL groups (*p* < 0.01), but not between AL and SJ groups (*p* = 0.353). Sdr demonstrated significant differences between SJ–CL and AL–CL groups (*p* < 0.01), but insignificant difference between SJ and AL groups (*p* = 0.81). Finally, for Sci the ranking of the statistically significant differences was SJ > AL > CL (*p* < 0.01).

### 3.3. Bond Strength Testing

**Figure 3:**
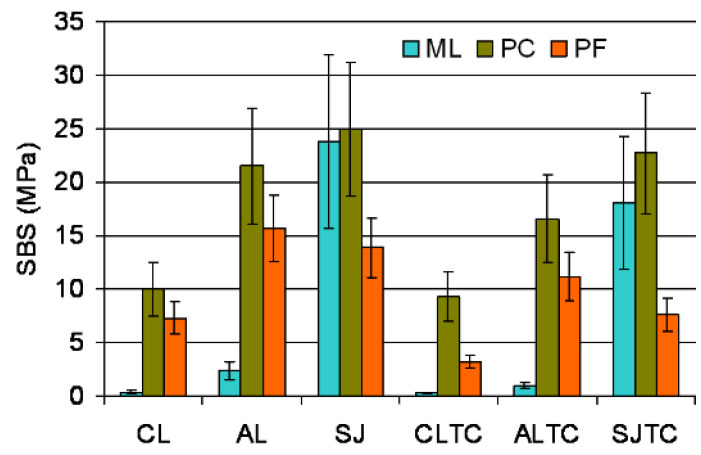
Means and standard deviations of the results of SBS.

**Figure 4:**
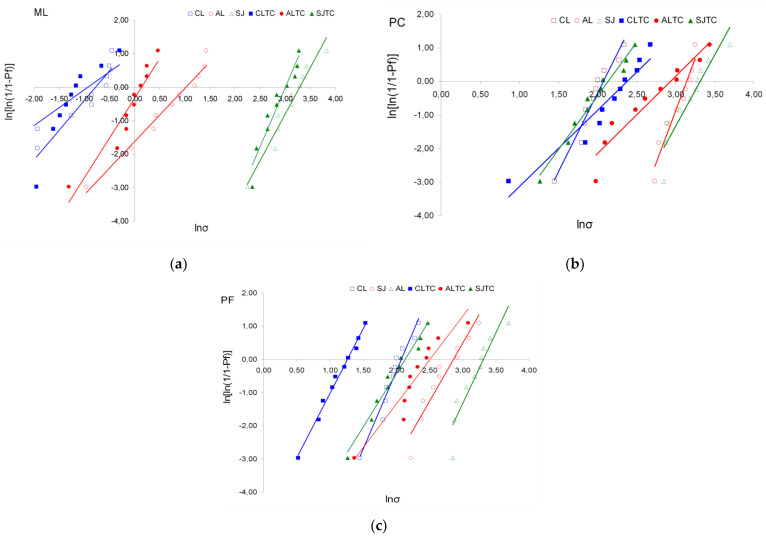
Weibull stress distribution graphs (ln/ln plots) of the various treatments per material. The slope of each graph (*vs y* axis) indicates the Weibull scale (m), while the intersection of the graph with the *x* axis indicates the Weibull modulus (σ_ο_, 63.2% fracture probability). (**a**) ML: Multilink Automix, (**b**) PC: PermaCem 2.0, (**c**) PF: Panavia F 2.0, CL: Control, AL: Alumina, SJ: Sil-Jet, TC: Thermal-cycling.

The descriptive statistics of shear bond strength testing (SBS) are illustrated in Figure 3. Weibull graphs of the SBS results for each material and testing condition are illustrated in Figure 4a–c; the numerical values for m, σ_ο_, 95% C.I. and r^2^ are presented in Table 3. The ranking of the statistically significant differences in σ_ο_ within each material subgroup were AL, SJ, ALTC > SJTC, CL > CLTC (PF); SJ, SJTC, AL, ALTC> CL, CLTC (PC) and SJ, SJTC > AL> ALTC > CL, CLTC (ML). Within the surface treatment subgroups, the ranking of the significant differences in σ_ο_ between the materials was PC, ML > PF (before and after TC) for SJ, PC > PF > ML (before TC) and PC, PF > ML (after TC) for AL, and PC > PF > ML (before and after TC) for CL. From the materials applied on grit-blasted surfaces, PF and ML were significantly affected by TC (SJTC and ALTC subgroups respectively). In PC, three subgroups manifested σ_ο_ > 20 ΜPa, two in ML and none in PF. For the m values, the only significant difference within each material group was found in PC (AL > ALTC) and for the same treatment in AL (PC > ML). In PF and PC three groups demonstrated m > 4 and none in ML.

**Figure 5:**
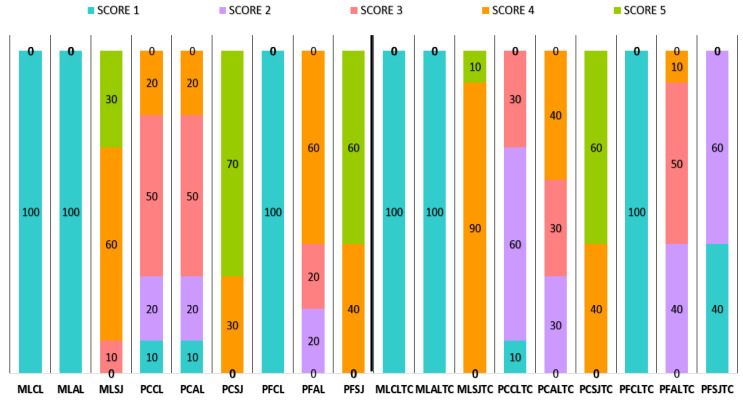
Failure mode percentage distribution for each material, surface treatment and storage condition (before and after thermal-cycling/TC).

The results of the failure mode analysis are presented in Figure 5. Adhesive failures (score 1) occurred in MLCL, MLAL and PFCL groups under isothermal testing and after thermal-cycling. Pearson’s Chi-square analysis showed that SJ treatment in ML group provided significantly higher score than CL and AL groups. In PF group, SJ and AL treatments presented similar scores, except for ALTC and SJTC, which were significantly different. In PC group, PCSJ, PCSJTC and PCAL were significantly different from all other subgroups.

## 4. Discussion

According to the results of the present study the null hypotheses (a) must be fully rejected for the extent of water plasticization for PF and partially for PC (1 w, 3 w), ML (3 w), (b) for all the roughness parameters after AL and SJ treatments vs. the control (CL) and (c) for the σ_o_ values of the SBS, since significant differences were found between the surface treatments within the same resin composite luting agent (CLA) group and within the same treatment between the CLA groups. For the m values of the SBS, rejection applied for all groups, except for ALTC, and the failure mode, since each CLA type was influenced by surface treatments according to the Chi-square analysis.

In the present study, three commercially available CLA with different compositions were selected, to include in the study representative materials from the types currently available. ML is free of adhesive monomers, PF contains the adhesive monomer 10-MDP, whereas, in the self-adhesive cement PC, 10-MDP and maleic ester monomers are incorporated. ML was chosen to investigate the influence of the mechanical retention, since poor chemical adhesion was expected in groups MLCL and MLAL. For the tribo-chemical treatment (SJ), a silane coupling agent was used. All the cements tested were evaluated in the delayed light-curing mode, which has been shown to improve cement properties [29,30,31]. A typical hardness test has been used to evaluate the extent of water-plasticization, with the comparisons limited to the effect of storage conditions per material. For roughness measurements two amplitude (Sa, Sz), one hybrid (Sdr) and one functional were performed.

(Sci) parameters were measured for a more precise description of the topographical changes after grit-blasting procedures [28]. No dentine primers were applied in the bond strength study (PF, ML) since the design of the present study aimed at testing the zirconia–cement interface. Apparently, any positive or negative effect of the acidic dentine primers in material setting has been ruled out. A short thermal-cycling period was used as an aging condition (ISO 2013) [32], which may reveal the early hydrolytic susceptibility of the interfaces tested. Such information is missing, since the extended thermal-cycling periods used cannot discriminate the onset of the hydrolytic degradation process on bond strength. To isolate the role of temperature fluctuations, comparisons were made between the thermal-cycled (TC) and the isothermally stored groups immersed in water for the same period. Finally, a macro-shear test was chosen, instead of the more popular micro-shear, since low strength values were expected in the MLCL and MLCLTC groups used as control. For such cases micro-shear tests are not indicated [33]. On the other hand, the inherent limitation of subsurface loading of the bonding substrate by interfacial macro-shear tests [34] does not seem to affect the results of the present study, since the high-strength zirconia base is insensitive to cohesive failures.

The hardness measurements revealed a significant VHN reduction of PF in water, at all the time intervals tested, whereas for PC and ML a significant reduction was noticed after 1 w (PC) and 3 w (PC and ML) water storage. This indicates an inherent water sensitivity of PF from the early immersion period, which could be assigned to the setting behavior of this material. Although PF is considered as a dual-cured material, the contribution of the self-curing mechanism seems to be weak. For this reason, it has been postulated that the self-etch primers of PF are essential for adequate self-curing of the cement [35,36]. However, applying a water-containing dentine primer on zirconia surfaces may create problems, since most zirconia-and universal-primers are water-free. An interesting finding was that PF demonstrated the water plasticization effect from the initial step (1 h) of the water immersion periods as opposed to the self-adhesive PC. This implies that the structure of the set material was more prone to water plasticization than PC, although the latter contained two sources of polar hydrophilic adhesive monomers (10-MDP, maleic acid ester). Differences in the degree of C=C conversion, network crosslinking density, filler content and acidic monomer neutralization capacity (i.e., by filler particles) may explain this finding. ML showed a significant VHN reduction only after 3 w water storage. This material, with the lowest filler content but free of acidic adhesive monomers, exhibited the least susceptibility of water plasticization among the materials tested, although it contained a hydrophilic comonomer (2-HEMA). Apparently, the presence of acidic monomers immobilized into the bulk polymer network of PC and PF may enhance water uptake, acid ionization and exertion of the plasticization effect.

Grinding of zirconia specimens with SiC papers up to 600 grit-size aimed to homogenize initial specimen roughness, since the as-sintered specimens may show texture variability caused by the CAM grinding conditions and sintering procedures used. A zirconia surface polished to high luster would further minimize micromechanical retention, revealing more efficiently the surface treatment effects. However, highly polished zirconia does not correspond to clinically relevant conditions for resin bonding. The Sa values measured in the CL group of the present study were similar to those of as-sintered restorations prepared by CAD/CAM devices [37]. The grit-blasting treatments tested (AL, SJ), increased the amplitude (Sa, Sz), hybrid (Sdr) and functional (Sci) roughness parameters in comparison with the control (CL). SJ presented significantly higher Sci value from AL. Apparently, the softer and smaller in size SJ particles from AL may create smaller in amplitude and area defects, capable of more core volume retention in comparison with AL and CL zirconia surfaces. The Sci and Sdr values recorded are in agreement with the findings of a recent study [38]. A six times increase in the grit-blasted surface area (AL, SJ) was documented by the Sdr values, facilitating thus the bonding procedures by increased micromechanical retention and chemical bonding to alumina and silica.

In groups without grit-blasting (CL), significant differences in SBS were found among the CLA tested; PC manifested significant higher σ_ο_ than PF and ML before and after TC. The ML, serving as a control of micromechanical retention, showed very low values, in agreement with a previous study [8]. This suggests that the use of the non-acidic hydrophilic co-monomer 2-HEMA in ML had a negligible effect on zirconia bonding. For PC, insignificant differences were found before and after TC, implying a stable chemical bonding, contrary to PF, which showed a significant strength reduction after the short TC procedure. Both these products contain 10-MDP with a proven chemical bonding capacity to zirconia [10]. In PC, the presence of a carboxylic monomer may provide a synergistic bonding mechanism, since it may react with zirconia forming a chelating bidentate zirconium carboxylate [39]. Other structural differences such as filler content, degree of C=C conversion, viscosity and porosity (hand-mix vs. automix) may also affect SBS and failure mode. The adhesive failure modes documented for all CL specimen groups show that the strength of the chemically bonded interfaces with minimal mechanical retention capacity were much weaker than the cohesive strength of the materials.

Alumina grit-blasting significantly increased roughness parameters and is expected to exert a positive effect on SBS in all CLA. Nevertheless, the net contribution of micromechanical retention (without chemical adhesion) was minimal and was further reduced after TC, as documented in MLCL and MLCLTC groups. The SBS values of PF and PC were significantly higher than ML, because of the establishment of interfacial bonding conditions on the rough substrate of zirconia and alumina. Alumina fragments implanted in zirconia surface have been identified after alumina grit-blasting or tribo-chemical coating procedures [12,13], where phosphate monomers can bond chemically [40]. The statistically insignificant differences in SBS between PC and PF on AL before and after TC, imply that the carboxylic monomers in PC do not substantially contribute to the SBS, especially when these cements demonstrated significant differences in the controls (CL). The adhesive failure modes on AL surfaces were modified in PF and PC in favor of adhesive failures, indicating a stronger bonding condition. In most specimens these failures were located opposite to the shear loading direction, due to the bending moments induced by the stress distribution.

Tribo-chemical silica coating was performed employing a commercially available silica–alumina powder system with a universal silane primer. The direct application of the CLA on silica–alumina grit-blasted surfaces was not evaluated, since phosphate and carboxylic monomers do not bond to silica as efficiently as the silanes [23]. On the other hand, a universal silane primer was selected (silane, phosphate and disulfide monomer components), to avoid coverage of the entire grit-blasted surface only with a silane layer. As the silica particle implantation pattern in zirconia is not uniform [23], the silanols are expected to selectively chemisorb onto silica domains via siloxane bonds, providing a weak physisorption pattern on alumina and zirconia regions, mostly limited to H-bonding. This may block alumina and zirconia sites available for bonding with phosphates. By using a silane/phosphate primer it is anticipated that the chemical affinity of the components to the substrate will be simultaneously mediated, accordingly. Moreover, the use of the two active components (silane, phosphate) in a liquid primer form, diminished the possibility of inadequate penetration into the rough surface texture of the adhesive monomers.

The SBS and failure mode results documented a significant positive impact of tribo-chemical coating on ML and PC before and after TC, but not on PF. The effect cannot be attributed to the side-interactions documented for silanes containing or combined with phosphate monomers like 10-MDP [14], since PC has a similar phosphate chemistry. A possible explanation might be the limited curing capacity of PF at regions distal to the activating light, mainly set by the self-curing mode, in the absence of primers [41]. Such conditions may reduce the stress absorption capacity of the cement specimens during loading.

Bond strength values between zirconia and CLA exhibit high variability [2]. The different types of zirconia, experimental setup (tensile, shear, etc.), specimen size, variety of materials and processing techniques, as well as storage conditions, are some of the variables rendering direct comparisons among different studies difficult or invalid. In the present experimental setup, three CLA types were tested in combination with three surface treatments, including controls of micromechanical retention. These in vitro results may enlighten the CLA bonding mechanisms involved, their early hydrolytic susceptibility and their specificity for the surface treatments tested.

## 5. Conclusions

Within the limitations of the present study, the following conclusions can be reached:

1. Hardness measurements showed that the adhesive resin composite luting agent demonstrated the earliest susceptibility to water plasticization, followed by the self-adhesive luting agent and the adhesive-free luting agent, the latter being the least affected.
2. Zirconia surface roughness parameters were significantly increased after alumina particle grit-blasting and tribo-chemical silica coating treatments.
3. Tribo-chemical silica coating combined with a silane coupling agent containing phosphate/disulfide monomers was the most efficient bonding treatment for the non-adhesive and the self-adhesive luting agents.
4. The adhesive luting agents were the best treatments for alumina grit-blasted zirconia.

## Figures and Tables

**Table 1:** The resin composite luting agents used in the study.

Product (Lot/Code)	Composition *	Manufacturer
Multilink Automix 532906/ML Shade: Yellow	EBPDMA, BisGMA, 2-HEMA, UDMA, catalysts, F-Ba-glass, Ba-Al-F-Silicate glass, YF_3_ (Filler: 66%wt, 40%v, size: 0.25–3 μm)	Ivoclar Vivadent, Schaan, Liechtenstein
Panavia F 2.0 2.000555A,00107B/PF Shade: A2	Hydrophobic aromatic and aliphatic dimethacrylates, hydrophilic aliphatic dimethacrylate, 10-MDP, catalysts, silanated silica, Ba-glass, colloidal silica, functionalized NaF (Filler: 78%wt, 59%v, size: 0.04–19 μm)	Noritake Kuraray, Osaka, Japan
PermaCem 2.0 20730226/PC Shade: A2	EBPDMA, BisGMA, TEGDMA, TMPTMA, 10-MDP, maleic acid ester, catalysts, Ba-glass, pyrogenic silica (Filler: 69%wt, 55%v, size: 0.02–3.0 μm)	DMG, Hamburg, Germany

* According to the SDS files of the manufacturers. EBDMA: Ethoxylated bisphenol-A dimethacrylate, BisGMA: Bisphenol-A glycidyl dimethacrylate, TEGDMA: Triethylene-glycol dimethacrylate, TMPTMA: Trimethylolpropane trimethacrylate, 10-MDP: 10-methacryloyloxydecyl dihydrogen phosphate, UDMA: Urethane dimethacrylate.

**Table 2:** Mean values and standard deviations of the surface roughness parameters tested. Same superscripts indicate mean values with no statistically significant differences per parameter (*p* > 0.05).

Groups	Sa (μm)	Sz (μm)	Sdr (%)	Sci
CL	0.31 (0.06) ^a^	2.57 (0.36) ^a^	4.2(1.1) ^a^	1.25(0.15) ^a^
AL	0.69 (0.13) ^b^	6.03(1.10) ^b^	24.7(1.8) ^b^	1.47(0.05) ^b^
SJ	0.66 (0.10) ^b^	6.34(0.63) ^b^	23.3(0.9) ^b^	1.61(0.09) ^c^

**Table 3:** Results of the Weibull modulus (m) and scale (σ_o_) parameters, with the corresponding 95% confidence intervals (C.I.) and the regression coefficient (r^2^) for materials, treatments and storage conditions (before and after thermal-cycling/TC) Same superscripts indicate values with no statistically significant difference (*p* > 0.05) within the same treatment between the cements (upper case) and the various treatments for the same cement (lower case).

Subgroups	m	m (95% C.I.)	σ_ο_(MPa)	σ_ο_ (95% C.I.)	r^2^
ML					
CL	2.2	1.3–3.8 ^A,a^	0.5	0.3–0.6 ^C,d^	0.87
CLTC	2.5	1.5–4.2 ^A,a^	0.4	0.3–0.5 ^C,d^	0.97
AL	1.9	1.2–3.3 ^B,a^	2.7	1.9–3.8 ^C,b^	0.97
ALTC	3.2	1.9–5.3 ^A,a^	1.1	0.9–1.3 ^B,c^	0.91
SJ	2.6	1.7–4.2 ^A,a^	26.8	20.9–34.3 ^A,a^	0.95
SJTC	3.7	2.2–6.0 ^A,a^	20.1	16.8–24.1 ^A,a^	0.98
PC					
CL	4.3	2.7–6.8 ^A,a,b^	11.0	9.4–12.8 ^A,b^	0.97
CLTC	3.1	1.9–5.3 ^A,a,b^	10.4	8.5–12.8 ^A,b^	0.91
AL	7.5	4.5–12.7 ^A,a^	23.0	21.0–25.0 ^B,a^	0.95
ALTC	2.3	1.4–3.8 ^A,b^	18.8	14.1–24.9 ^A,a^	0.98
SJ	3.9	2.5–6.0 ^A,a,b^	27.5	23.2–32.6 ^A,a^	0.95
SJTC	4.7	2.9–7.6 ^A,a,b^	24.8	21.6–28.5 ^A,a^	0.96
PF					
CL	4.6	2.9–7.4 ^A,a^	7.9	6.9–9.1 ^B,b^	0.94
CLTC	4.1	2.5–6.7 ^A,a^	3.5	3.0–4.1 ^B,c^	0.99
AL	3.3	2.1–5.3 ^A,a^	17.5	14.4–21^A,a^	0.98
ALTC	2.8	1.8–4.3 ^A,a^	12.5	10.1–15.8 ^A,a^	0.96
SJ	4.9	3.0–7.9 ^A,a^	15.1	13.2–17.3 ^B,^^a^	0.95
